# Monitoring of essential and heavy metals in green tea from different geographical origins

**DOI:** 10.1007/s10661-016-5157-y

**Published:** 2016-02-22

**Authors:** Justyna Brzezicha-Cirocka, Małgorzata Grembecka, Piotr Szefer

**Affiliations:** Department of Food Sciences, Medical University of Gdansk, Al. Gen. J. Hallera 107, Gdansk, 80-416 Poland

**Keywords:** Green tea, Heavy metals, PTWI, Factor analysis, Cluster analysis

## Abstract

The present study measured the concentrations of toxic metals (Cd, Pb) and other elements (Ca, K, Mg, Na, P, Mn, Fe, Zn, Cu, Co, Cr, Ni) in tea leaves and their infusions. The total metal contents were determined by atomic absorption spectrometry. Phosphorus concentration was determined using an ultraviolet–visible spectrophotometer. Assessment of the mineral composition enabled determination of the leaching percentage and the risk of exceeding provisional tolerable weekly intake for Cd through daily tea consumption. The concentrations of bioelements were analyzed based on the recommended daily intake values for each. According to recently established standards, green tea was found to be a rich source of Mn. The average Pb and Cd levels in a 200-mL beverage were 0.002 and 0.003 mg, respectively. Indian teas had the highest percentage of Cd leaching (43.8 %) and Chinese tea had the lowest (9.41 %). Multivariate analysis techniques such as factor analysis and cluster analysis were used to differentiate samples according to geographical origin (China, India, or Japan). Potassium, P, Mn, Fe, Cu, Co, and Cd were effective descriptors for the identification of tea samples from China, India, and Japan.

## Introduction

Tea is one of the most popular beverages in the world mainly because of its pleasant aroma, flavor, and potential positive effect on mood. It has the second position in the ranking of the most widely consumed beverages (Shen and Chen 2008). China is the leading producer of tea in the world, with 1,726,023 tons produced in 2011 (FAOSTAT 2014). Other countries producing large quantities of tea include India, Kenya, and Sri Lanka (FAOSTAT 2014).

*Camellia sinensis* is the main source of tea leaves, and the quality of tea depends on its cultivation conditions, the soil in which it grows, the degree of contamination, and numerous meteorological conditions.

Recently, there has been observed a growing interest in green tea. Tea antioxidant properties are due to various compounds that prevent Alzheimer’s disease, hypertension, and obesity (McKenzie et al. 2010). Besides many biologically active substances such as polyphenols and tannins (Szymczycha-Madeja et al. 2012), tea also contains bioelements as well as toxic metals such as cadmium (Cd) and lead (Pb). These two metals cause permanent damage to key enzymes and many systems of the body including the circulatory, renal, and central nervous ones (Santos et al. 2013). Therefore, it is important to monitor the concentration of these metals, especially in view of permissible limits for growth and good health.

Considering that tea is consumed as a beverage, the percentage of metals leaching into infusions needs to be estimated. Based on the mineral composition of teas, it is possible to estimate the permissible limits for heavy metals and the health hazards associated with their intake. Determining the provisional tolerable weekly intake (PTWI) for Cd is critical. Additionally, assessment of the composition of bioelements against their recommended dietary allowance (RDA) for an adult person is important information, not only for dietitians but also for consumers.

Assessment of the concentrations of elements in the different teas enabled their differentiation according to mineral composition and their classification according to geographical origin. It was performed using chemometric techniques that allow on confirmation of food authenticity in view of its composition (Herrador and González 2001; Fernández et al. 2002; Moreda-Piñeiro et al. 2003; Szefer 2007). Among many compounds that can be used to verify the origin and quality of food products are bioelements that usually reflect the soil composition as well as influence of environmental factors. They can also be used as markers of the level of technological processing which was applied to the products. In the literature, there can be found many examples of multivariate techniques of application to such food product analyses such as tea, fruits, honey, virgin olive oils, nuts (pistachios), and many others (Szefer 2007; McKenzie et al. 2010; Grembecka and Szefer 2013a, b; Karabagias et al. 2013; Sciubba et al. 2014).

In case of tea of one type, for instance green, it is important to find factors which will be helpful in its discrimination, i.e., that will reflect the variations resulting from different origins. Therefore, mineral composition is a better choice than organic compounds, which mainly depend on technological processing, and are more suitable to differentiate particular kinds of tea, i.e., green from black (Moreda-Piñeiro et al. 2003).

Herrador and González (2001), Moreda-Piñeiro et al. (2003), and McKenzie et al. (2010) studied mineral composition of tea using modern chemometric methods, which confirmed the differentiation of individual types of teas. Herrador and González (2001) differentiated black, green, and oolong teas by principal component analysis (PCA), cluster analysis, and linear discriminant analysis (LDA) and artificial neural networks trained by back propagation. McKenzie et al. (2010) also conducted chemometrical analysis of various kinds of tea, i.e., white, green, black, oolong, and Pu-erh, based on their mineral composition. Moreover, Moreda-Piñeiro et al. (2003) confirmed, using multivariate techniques that the classification of teas originating from Africa and Asia in view of their country of origin and authenticity is also possible.

In our study, there were also applied chemometric techniques, which were designed to diversify samples of one type of tea, i.e., green tea, and it was possible to confirm the geographical origin of samples based on their mineral composition. Such research is becoming more and more important in view of the introduction of the Regulation of the European Parliament and of the Council (EU) No 1169/(2011) of 25 October 2011 on the provision of information to consumers about food, which requires country of origin information on the product label (Regulation (EU) No 1169/2011). However, few manufacturers provide this information.

In the present study, we analyzed and compared the concentrations of mineral nutrients and toxic metals in 41 green teas, including their infusions, from different geographic regions (China, India, Japan, and others) obtained from the local market. The health benefits and hazards associated with green tea consumption were estimated in view of permissible dietary limits. Evaluation of elemental composition allowed on quantitative differentiation of green tea samples in view of their geographical provenance.

## Materials and methods

### Samples

All teas in loose form and tea bags were chosen to represent the variety of green tea products available on the market in Poland. They were purchased from various tea shops (tea of certified origin) and markets (marketed tea). The characteristics of green tea species are summarized in Table 1. In total, 41 types of green tea were tested and approximately 500 analytical samples were prepared.

**Table 1:** Characteristics of the analyzed products

Lp.	Name of tea	Producer	Country/producer declaration	Confection
Original tea				
1.	Ch. Green Yunnan	Time to tea	China	Loose
2.	Yunnan Green	Five’oclock	China	Loose
3.	China Yunnan	Maraska	China	Loose
4.	ChunMee^a^	Manhattan	China	Loose
5.	ChunMee^a^	Maraska	China	Loose
6.	Grunpowder TOH^a^	Five’oclock	China	Loose
7.	Grunpowder TOH^a^	Manhattan	China	Loose
8.	Grunpowder TOH^a^	Maraska	China	Loose
9.	Dajreeling Arya	Five'oclock	India	Loose
10.	Darjeeling Green	Time to tea	India	Loose
11.	Japan Bancha^a^	Five’oclock	Japan	Loose
12.	Japan Sencha^a^	Time to tea	Japan	Loose
13.	Japan Bancha^a^	Maraska	Japan	Loose
14.	Japan Kokeicha	Five o’clock	Japan	Loose
15.	Kokeicha	Manhattan	Japan	Loose
16.	Japan Sencha^a^	Manhattan	Japan	Loose
17.	Japan SenchaFukuyu	Five’oclock	Japan	Loose
Marketed tea				
18.	Green—Pure Green	Irving	China	Bags
19.	Green Grunpowder	Malwa	China	Loose
20.	Green Original China Tea	Sir Roger	China	Loose
21.	Green TeaSencha	Sir William’s	China	Bags
22.	Green Tea^a^	Teekanne	China	Bags
23.	Green Tea^a^	Biofix	China	Loose
24.	Green Tea^a^	Yunnan	China	Loose
25.	Green Tea^a^	Malwa	China	Bags
26.	Green^a^	Irving	China	Loose
27.	Pure Green Tea	Twinings of London	China	Loose
28.	Zen Chai	Teekanne	China	Bags
29.	Green TeaOrganic	Darvilles of Windsor	India	Bags
30.	Natural Green Tea	Tarlton	India	Bags
31.	Intensive Green Tea	Telety	Kenia	Bags
32.	Green Tea Pure Green	Dilmah	Sri Lanka	Bags
33.	Ceylon Green Tea	Dilmah	Sri Lanka	Bags
34.	Green Tea Nature	Lipton	–	Bags
35.	Grunpowder Green Tea	Twinings	–	Loose
36.	Green Tea^a^	Posti	–	Loose
37.	Leafy Green Tea	Vitax	–	Loose
38.	Green Tea^a^	Saga	–	Bags
39.	Green Tea^a^	Vitax	–	Bags
40.	The Green Leaf	Herbapol	–	Loose
41.	Green^a^	Herbapol	–	Bags

^a^various producers of tea under the same name

### Preparation of samples

The procedure concerning preparations of samples was adopted from Grembecka et al. (2007) with modifications. Firstly, tea was homogenized, using an electric grinder (IKA^®^ A-11 basic), and then 10.0-g (±0.0001 g) portions were weighed and transferred to quartz crucibles. The first part of mineralization was conducted in an electric furnace using a temperature gradient with a maximum temperature of 540 °C. Subsequently, 1.50 mL of 36 % HCl (Tracepure, Merck, Darmstadt, Germany) and two to three drops of 63 % HNO_3_ (Tracepure, Merck) were added to ashes and evaporated to dryness on a water bath. Then, again, 1.50 mL of 36 % HCl was added to residues, which underwent evaporation for 1 min under a watch glass. Afterward, the resulting solution was transferred to 25-mL flasks with ultrapure water (18 MΩ cm^−1^) from a Mili-Q system (Millipore, MA, USA).

Tea infusions were prepared using 2.00 g (±0.0001 g) of material and 200 mL of water in a 250-mL beaker. Tea was infused for 5 min and then it was filtered through a fluted paper filter. The resulting filtrate, which was transferred to a quartz crucible, was evaporated to dryness on a boiling water bath and the dry residue was ashed in a furnace following the same procedure as that used for the dry sample (Grembecka et al. 2007).

### Analyses of elements

The concentrations of elements (Ca, K, Mg, Na, P, Mn, Fe, Zn, Cu, Co, Cd, Cr, Ni, Pb) were determined by atomic absorption spectrometry with flame air-acetylene using the deuterium background correction. The analysis was performed using Thermo Scientific’s i3000. Cesium chloride (Cesium chloride, Merck), which acts as an ionizing buffer that shifts the equilibrium of the reaction to generate free atoms of the analyzed element (0.2 % *w*/*v*), was added to the samples in order to analyze K and Na content (Grembecka et al. 2007). Ca and Mg were analyzed by addition of 0.4 % *w*/*v* lanthanum oxide (Lanthanum (III) oxide, Merck), which is a correction buffer that allows binding of the analyzed element to a matrix. Phosphorus was determined using a spectrophotometric method (AOAC 2002) with an ultraviolet–visible spectrophotometer (Spekol 11, Carl Zeiss, Jena, Germany) .

The limits of detection (LODs) and the limits of quantification (LOQs) of the applied method were calculated using formulas proposed by Konieczka and Namieśnik (2009), i.e., LOD = blank mean + 3 SD and LOQ = 3 × LOD (Table 2) (Grembecka and Szefer 2013a). The reproducibility of the method was assessed using the certified reference material (tea, NCS ZC73014), which was digested using the same procedure as that used for tea samples. The results of the measurement of toxic metals and bioelements in the reference material are summarized in Table 2, which shows that the estimated element recovery and RSDs were satisfactory.

**Table 2:** Validation data of the analytical methodology

Element	Linearity			LOD (mg/100 g)	LOQ (mg/100 g)	RSD (%)	Recovery (%)
	Calibration curve range (μg/mL)	Calibration curve	R^2^				
Ca	2.00–15.0	*y* = 0.05801*x* + 0.0089	0.999	0.020	0.060	4.42	86
K	0.50–1.50	*y* = 0.00048*x* + 0.0149	0.997	0.040	0.120	4.81	102
Mg	0.10–0.90	*y* = 0.00107*x* + 0.0220	0.998	0.020	0.060	1.60	96
Na	0.50–1.20	*y* = 0.00071*x* + 0.0192	0.996	0.020	0.060	10.0	98
P	0.10–1.20	*y* = 0.00444*x* + 0.0117	0.999	0.030	0.090	0.11	103
Mn	0.15–5.00	*y* = 0.00015*x* + 0.0058	0.999	0.020	0.060	0.87	92
Fe	1.00–10.0	*y* = 0.00006*x* + 0.0082	0.996	0.010	0.030	5.73	101
Zn	0.20–1.50	*y* = 0.00034*x* + 0.0079	0.998	0.020	0.060	0.51	100
Cu	0.50–4.00	*y* = 0.00013*x* + 0.0022	0.999	0.009	0.027	0.12	91
Co	1.00–5.00	y = 0.00008*x* + 0.0052	0.999	0.003	0.009	11.1	95
Cd	0.20–2.00	y = 0.00035*x* + 0.0040	0.999	0.003	0.009	10.3	97
Cr	0.20–2.00	*y* = 0.00005*x* + 0.0007	0.999	0.001	0.003	4.51	98
Ni	0.50–2.00	*y* = 0.00008*x* + 0.0007	0.999	0.002	0.006	0.62	94
Pb	0.20–2.00	*y* = 0.00004*x* + 0.0004	0.999	0.004	0.012	2.11	94

### Statistical analysis

Decision of the type of statistical analyses used, parametric and non-parametric, was made after application of the normality Shapiro–Wilk test (Szefer 2007). As a result, there were chosen non-parametric tests, i.e., the Spearman rank correlation and the Kruskal–Wallis test as well as factor analysis (FA) and cluster analysis (CA). All the calculations were made with the use of Statistica 10 program. All data were standardized and then elements were set as columns and tea samples as rows in the created data matrix.

The Kruskal–Wallis test and a series of preliminary FAs resulted in the elimination of elements from the dataset because of their negative impact on the diversification of samples in the final analysis. Finally, seven elements (K, P, Mn, Fe, Cu, Co and Cd) were loaded onto the data matrix. In the CA, the best results were obtained with Ward’s method using Euclidean distance.

## Results and discussion

The concentrations of bioelements and toxic metals in the tea samples and percentages of leaching are listed in Table 3.

**Table 3:** Concentration of bioelements and toxic metals in tea samples in mg/100 g ($\overline{x}\pm \mathrm{S}\mathrm{D}$, range) and percent of leaching (%)

Tea	n	Ca	K	Mg	Na	P	Mn	Fe
Original tea								
China	8 × 3	82.5 ± 61.2	2006 ± 335	225 ± 20.3	4.77 ± 3.20	337 ± 68.1	66.5 ± 22.8	33.6 ± 12.7
		(21.6–231)	(1579–2636)	(193–256)	(3.11–13.2)	(235–445)	(30.9–96.5)	(15.0–56.2)
		33.6 ± 24.0 %	59.0 ± 12.4 %	40.0 ± 9.36 %	18.1 ± 8.95 %	30.8 ± 4.51 %	31.4 ± 2.01 %	7.89 ± 3.53 %
India	2 × 3	74.8 ± 48.1	2215 ± 44.6	239 ± 17.1	2.34 ± 0.23	365 ± 44.9	39.0 ± 8.29	15.4 ± 0.09
		(26.7–123)	(2171–2260)	(222–256)	(2.11–2.57)	(320–410)	(30.7–47.3)	(15.3–15.5)
		22.2 ± 3.14 %	66.3 ± 10.7 %	35.5 ± 3.21 %	43.7 ± 0.16 %	40.1 ± 11.1 %	30.0 ± 0.19 %	13.4 ± 0.13 %
Japan	7 × 3	83.0 ± 38.1	1859 ± 261	214 ± 21.9	8.80 ± 7.21	236 ± 46.6	126 ± 11.0	33.2 ± 17.0
		(51.7–169)	(1503–2216)	(195–262)	(2.00–24.7)	(155–307)	(106–138)	(19.5–60.1)
		29.2 ± 16.0 %	54.2 ± 18.9 %	26.8 ± 6.11 %	24.2 ± 17.1 %	22.5 ± 8.91 %	24.2 ± 2.94 %	7.85 ± 2.61 %
Marketed tea								
Marketed	24 × 3	94.7 ± 46.1	1997 ± 517	243 ± 43.3	8.73 ± 7.04	282 ± 41.2	96.2 ± 34.3	31.0 ± 13.0
		(29.9–230)	(1552–4281)	(155–326)	(1.78–49.6)	(221–387)	(24.8–160)	(12.8–59.7)
		16.9 ± 7.54 %	58.5 ± 13.3 %	37.9 ± 14.0 %	15.1 ± 12.1 %	28.9 ± 13.7 %	29.3 ± 6.00 %	8.31 ± 3.08 %
Tea	n	Zn	Cu	Co	Cd	Cr	Ni	Pb
Original tea								
China	8 × 3	3.69 ± 1.14	1.97 ± 0.30	0.03 ± 0.01	0.01 ± 0.004	0.10 ± 0.04	0.82 ± 0.28	0.73 ± 0.36
		(1.17–4.94)	(1.63–2.59)	(0.01–0.04)	(0.003–0.01)	(0.04–0.14)	(0.37–1.22)	(0.22–1.32)
		46.3 ± 21.6 %	24.4 ± 7.46 %	54.9 ± 22.3 %	9.41 ± 5.44 %	36.5 ± 21.5 %	38.0 ± 7.34 %	16.9 ± 14.3 %
India	2 × 3	3.84 ± 0.03	1.77 ± 0.12	0.01 ± 0.001	0.003 ± 0.0005	0.10 ± 0.03	0.60 ± 0.06	0.1 ± 0.07
		(3.81–3.87)	(1.65–1.89)	(0.01–0.01)	(0.003–0.004)	(0.03–0.10)	(0.55–0.66)	(0.009–0.14)
		39.4 ± 1.00 %	24.6 ± 1.27 %	49.6 ± 20.3 %	43.8 ± 1.31 %	79.0 ± 16.9 %	34.2 ± 5.31 %	39.3 ± 9.20 %
Japan	7 × 3	3.11 ± 0.84	1.34 ± 0.08	0.05 ± 0.01	0.006 ± 0.001	0.16 ± 0.11	0.52 ± 0.10	0.84 ± 0.32
		(1.52–4.23)	(1.23–1.50)	(0.03–0.06)	(0.003–0.01)	(0.10–0.34)	(0.39–0.70)	(0.51–1.50)
		38.7 ± 15.2 %	26.7 ± 4.86 %	67.4 ± 11.8 %	17.6 ± 9.3 %	21.2 ± 17.4 %	37.1 ± 5.42 %	12.6 ± 7.01 %
Marketed tea								
Marketed	24 × 3	4.07 ± 1.29	2.03 ± 0.45	0.03 ± 0.01	0.01 ± 0.004	0.10 ± 0.06	0.63 ± 0.25	0.45 ± 0.38
		(2.81–9.58)	(1.41–3.41)	(0.01–0.05)	(0.003–0.01)	(0.05–0.34)	(0.21–1.08)	(0.09–1.38)
		34.4 ± 6.54 %	27.0 ± 6.18 %	63.7 ± 20.6 %	24.3 ± 12.9 %	44.7 ± 18.0 %	40.6 ± 8.45 %	29.3 ± 26.0 %

*n* – number of samples multiplied by number of analytical subsample

### Toxic elements

Green tea from India had the lowest concentration of Cd (0.003 mg/100 g) and Pb (0.10 mg/100 g) among the samples analyzed. Han et al. (2005) reported higher results for Cd (0.01 mg/100 g). Moreda-Piñeiro et al. (2003) reported a concentration of Pb of 0.21 mg/100 g in Chinese teas, which was lower than the value obtained in the present study (0.73 mg/100 g). According to Santos et al. (2013), it can be explained by the variations in Pb contamination sources of anthropogenic provenance, i.e., batteries, paints, dyes, and heavy industries. Moreover, Souza (2005) implied that 96 % of lead in the atmosphere is of anthropogenic origin. What is more important, it was found that Pb is more bioavailable to tea plants growing in highly acidic soils (Han et al. 2006a, b). Shi et al. (2007) based on analysis of 328 tea samples for Cd (collected from the main tea-producing regions of China during the years 1997 and 1998) report 0.006 mg of Cd in 100 g. However, in 2004, the average Cd level increased to 0.01 mg/100 g, which is comparable with our results. Such increase in Cd levels can be explained by tea contamination both by its accumulation in plants during the growth period and the manufacturing processes. According to the available literature, tea infusions were characterized with low Cd contents (Karak and Bhagat 2010). Indian teas had the highest percentage of Cd leaching (43.8 %) and Chinese tea had the lowest (9.41 %). Japanese tea had the highest Pb contamination (0.84 mg/100 g) and the percentage of leaching of this heavy metal amounted to 12.6 % (Table 3). Few studies have assessed the concentration of Pb in tea infusions. Jin et al. (2005) observed a restricted leaching of Pb from tea leaves during soaking in boiling water. Karak and Bhagat (2010) suggested that there is a risk of exceeding the World Health Organization (WHO) limit set for Pb in drinking water (0.05 mg/L) by tea infusions (WHO 2003). Therefore, the contamination of tea leaves by Pb remains an issue of concern, and practices should be developed to avoid problems in the future.

### Macroelements

Among the macroelements, K was present at the highest concentrations, with values of 2215 mg/100 g in Indian tea and 2006 mg/100 g in Chinese tea, which is in agreement with the results published by Kumar et al. (2005), Li et al. (2007), and Soomro et al. (2008). Such high concentrations might be explained, as Kumar et al. (2005) suggested, by the specific incorporation of K within a binding ligand of the tea leaves. However, concentration of K in Japanese brands was in the range of 1503–2216 mg/100 g with a mean content of 1859 mg/100 g. According to Kumar et al. (2005), Indian and US tea brands contained between 1770–2400 and 1310–2370 mg K/100 g, respectively. Dambiec et al. (2013) found the lowest K content in tea from Argentina and Vietnam and the highest in samples from Central India. They reported that K concentration in tea is higher than Mg, while the Mg content is higher than Na one. The differences in results concerning macroelements’ contents obtained by various researchers depend on tea sample origins and conditions of their cultivations. According to Yemane et al. (2008), tea leaves, independently of their brand, contain higher levels of K than of Na. Moreover, Na content, within different brands of tea, shows large variability (Kumar et al. 2005). Sodium is characterized by low concentrations, especially in Japanese (8.80 mg/100 g) and marketed tea (8.73 mg/100 g), which is comparable with values (8.80 mg/100 g) reported by McKenzie et al. (2010). According to Kumar et al. (2005), Na content varies widely (2.1–11.8 mg/100 g) in Indian tea brands, with a mean value of 5.35 mg/100 g. However, US tea brands were characterized by a wider range of Na concentrations (11.4–79.6 mg/100 g) with a mean value of 33.8 mg/100 g. Kumar et al. (2005) explained that US tea brands characterize with higher Na levels partially due to flavoring additives.

On the other hand, Varnam and Sutherland (1994) suggested that green tea character mainly depends on leaf compositions at the time of harvesting than on compounds formed during technological processing. Moreover, the elemental composition of green tea is strongly associated with its geographical origin (Marcos et al. 1998; Fernández-Cáceres et al. 2001; Moreda-Piñeiro et al. 2003), genetic differences (Fung et al. 2003, 2009), soil composition, and agricultural or climatic conditions (Fung et al. 2003, 2009; Mehra and Baker 2007; Seenivasan et al. 2008b).

The determined percentage of extraction (Table 3) decreased in the following order: K (54.2–66.3 %), Mg (26.8–40.0 %), and P (22.5–40.1 %). Dambiec et al. (2013) also found that K is extracted to infusion to the greatest extent. They also noticed that percentage of leaching attributed to Na is the lowest among all the macroelements (Dambiec et al. 2013). However, in our study, Indian tea showed the highest percentage of extraction of Na (43.7 %), whereas for other types of tea, it ranged between 15.1 and 24.2 %.

### Microelements

Manganese was the most abundant microelement in the teas analyzed (39.0–126 mg/100 g), which confirms the suggestion made by Dambiec et al. (2013) that tea is a Mn accumulator. The concentration of Mn in marketed green tea (96.2 mg/100 g) was similar to that reported by Pohl and Prusisz (2007) (96.0 mg/100 g) and McKenzie et al. (2010) (105 mg/100 g). Indian tea had the lowest concentration of Mn (39.0 mg/100 g), Fe (15 mg/100 g), Co (0.01 mg/100 g), and Cr (0.10 mg/100 g). Pękal et al. (2013) reported similar levels of elements such as Fe (13.0 mg/100 g) and Cu (1.80 mg/100 g) for Indian green tea. Jin et al. (2008) and Mehra and Baker (2007) determined similar values for Cu in Chinese teas. The values reported by Mandiwana et al. (2011) for Cr in Chinese teas (0.07 mg/100 g) and Han et al. (2005) for Co (0.04 mg/100 g) were also comparable to those of the present study. Al-Othman et al. (2012) obtained comparable values for Ni (0.51 mg/100 g) in marketed tea. Somewhat higher and comparable percentages of leaching in all green teas were noted for Mn (24.2–31.4 %) and Cu (24.4–27.0 %), which is also in accordance with the results of Pohl and Prusisz (2007) for the same marketed teas bought in Poland (Mn 22.0 %). Indian tea had a higher percentage of Fe leaching (13.4 %) than teas of other origins (ca. 8.00 %). The low Fe percentage of leaching may be explained by formation of complexes of low solubility (Soomro et al. 2008). Moreover, tannic acid as well as tannins reacts with elements in tea leaves, which might result in tea infusions’ varied composition (Brzezicha-Cirocka et al. 2015; Powell et al. 1998). Precipitation of these chelates significantly decreases the concentration of metals in the brew extract. Dambiec et al. (2013) suggested that teas, which characterize with lower tannin levels, reveal better percentage of leaching of particular elements to infusions.

### Kruskal–Wallis test

There was applied non-parametric test, i.e., the Kruskal–Wallis test, which was used to evaluate the differences in mean concentrations between the tea groups from various geographical provenances in view of mineral composition (Herrador and González 2001; Fernández et al. 2002; Moreda-Piñeiro et al. 2003; McKenzie et al. 2010; Table 4). Based on the obtained results, it was concluded that elemental composition of tea is dependent on its country of origin. It was noticed that concentrations of such elements as P (H = 13.235; *p* = 0.001), Mn (H = 10.557; *p* = 0.005), Fe (H = 13.235; *p* = 0.013), Cu (H = 12.059; *p* = 0.002), Co (H = 8.809; *p* = 0.012), Cd (H = 6.271; *p* = 0.043), and Cr (H = 12.059; *p* = 0.002) are dependent on the geographical origin of samples. Comparable results of ANOVA Kruskal–Wallis test to our study were also obtained by McKenzie et al. (2010) who have reported that there are strong interdependences in tea samples for elements Mn, Cu, Fe, and P in view of geographical origin. In addition, Moreda-Piñeiro et al. (2003) found statistically significant differences between Fe, Mn, and Cu for tea samples from China and India (Sri Lanka). According to Moreda-Piñeiro et al. (2003), it is possible to classify tea samples in view of their geographical provenance based on their mineral composition.

**Table 4:** The influence of the geographical provenance on elemental composition in view of ANOVA Kruskal–Wallis test

Green Tea		Ca	K	Mg	Na	P	Mn	Fe	Zn	Cu	Co	Cd	Cr	Ni	Pb
Geographical origin	*p*	0.93	0.30	0.27	0.20	0.001	0.005	0.01	0.34	0.002	0.01	0.04	0.002	0.07	0.07
	H	0.15	2.42	2.61	3.22	13.2	10.6	13.2	2.17	12.1	8.81	6.27	12.1	5.32	5.26

*p* level of significance, *H* test value

### Factor analysis

**Fig. 1:**
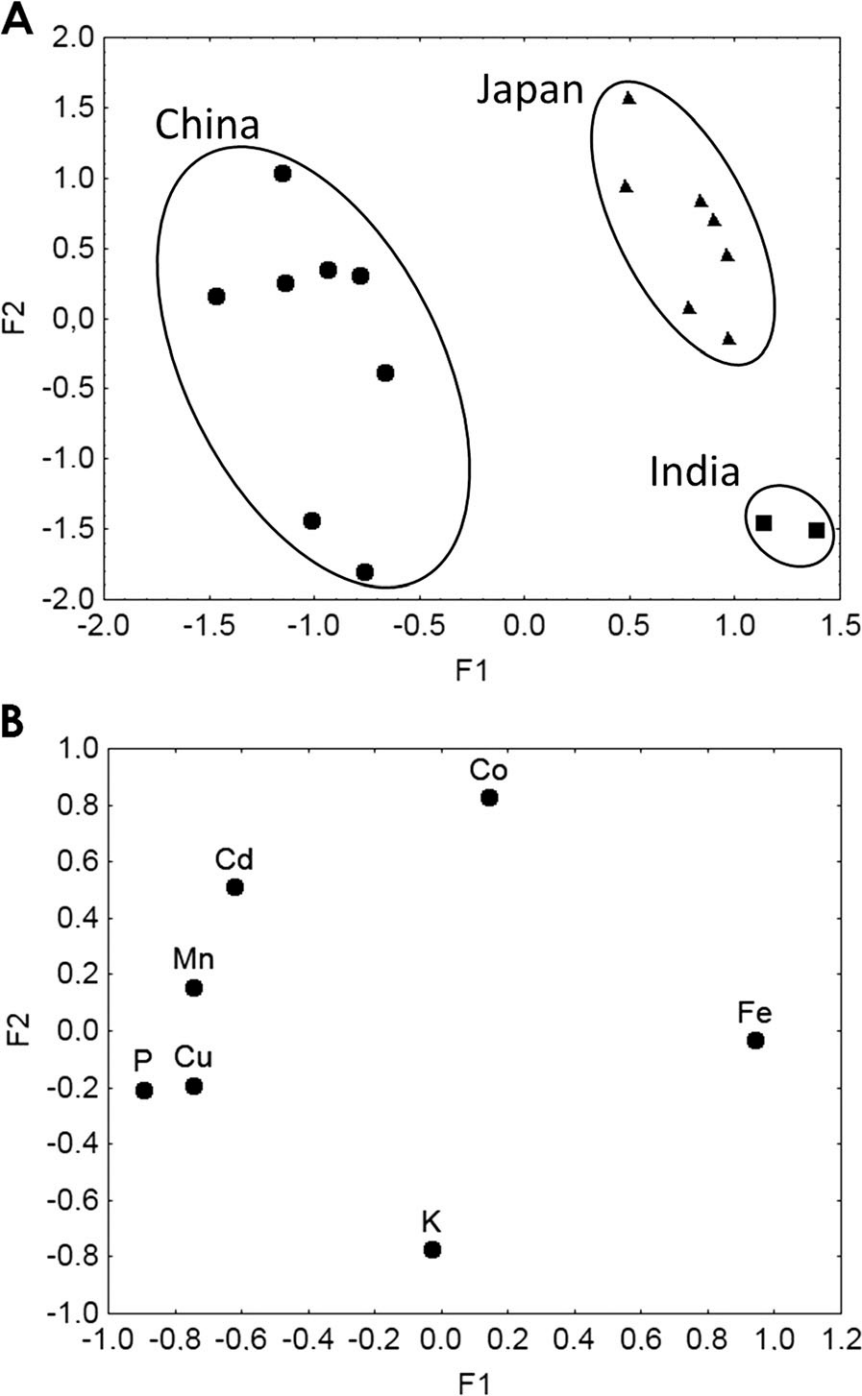
**a** Scatterplot of the factors distinguishing green tea samples from various geographical origins. **b** Scatterplot of loadings for seven elements in green tea samples from various geographical origins

Green tea purchased from tea shops appears to be appropriate ultimate datasets for FA, in which results are presented on Fig. 1a, b. There were two obtained factors, i.e., F1 and F2, that cumulatively explain 69.28 % of the total variance (F1 = 45.75 % and F2 = 23.53 %). The eigenvalues for F1 and F2 amounted to 3.20 and 1.64, respectively.

As shown in Fig. 1a, there were clear distinctions between samples from different regions such as China, India, and Japan. The scatterplot of loadings was drawn for F1–F2 to identify elements responsible for the grouping of objects (Fig. 1b). Higher F1 values for K, Fe, and Co corresponded to Japanese and Indian samples, whereas lower values for P, Mn, Cu, and Cd were characteristic of Chinese tea (Fig. 1a, b).

Regarding F2, samples were differentiated according to geographical origin mainly for Indian and Japanese teas. Lower F2 values were associated with Indian tea samples, and they overlapped partly with Chinese samples, which were identified mainly by their K content. Higher scores were obtained for Japanese samples (overlapped partly with Chinese samples), which were identified by their Co content (Fig. 1a, b).

### Cluster analysis

**Fig. 2:**
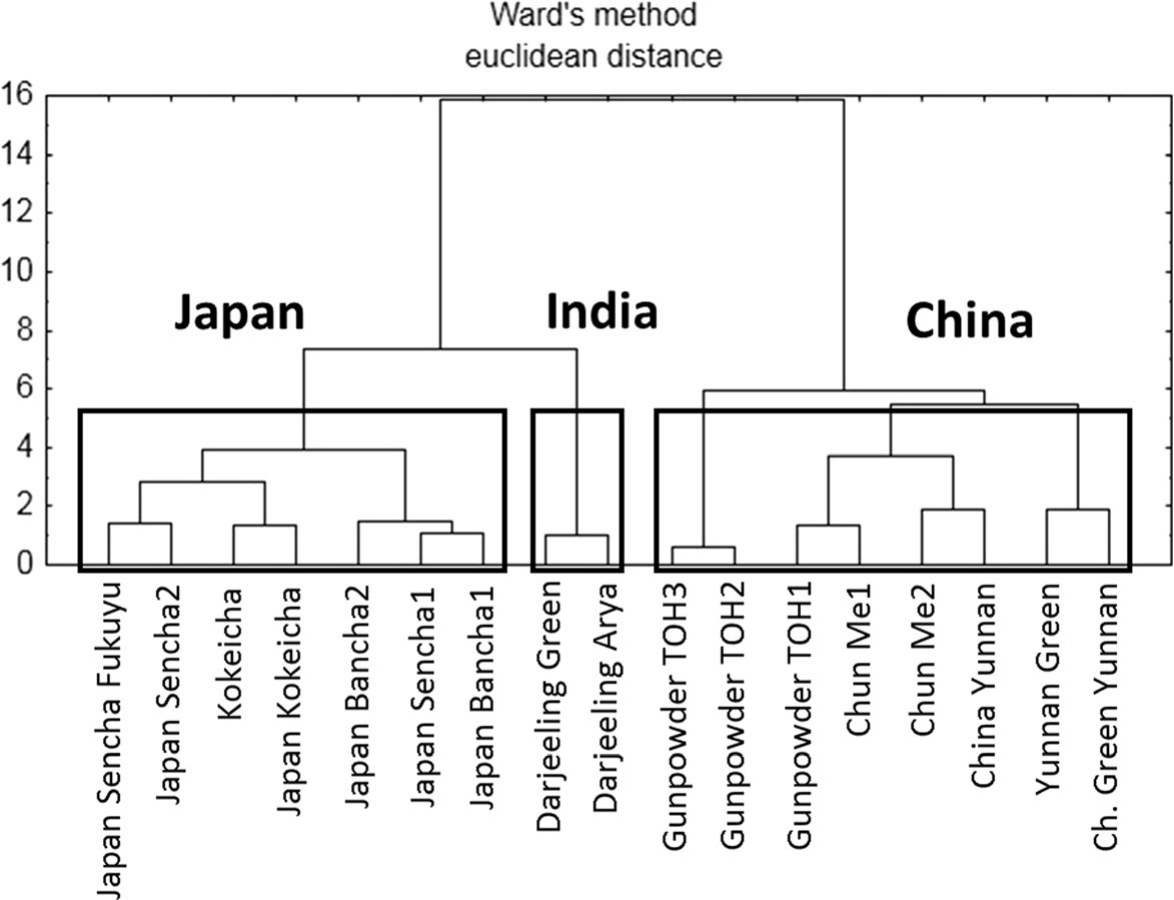
Hierarchical dendrogram of the analyzed original green tea samples as objects according to geographical origin

Figure 2 shows the dendrogram which was built with three main clusters containing samples from Japan, India, and China. As can be seen in this figure, CA enabled the differentiation of green tea samples according to geographical origin based on the chemical composition of the analyzed samples (Fig. 2).

These results suggest that CA, similarly to FA, can distinguish tea samples according to their geographical origin, which is important for the assessment of authenticity and fraud detection.

### Recommended dietary intake

The WHO/FAO (2010) committee has set the PTWI for Cd at 7 μg/kg, which recalculated for a person weighing 70 kg equals 490 μg/person. However, the recommendation (PTWI) made for Pb, which amounted to 25 μg/kg of body weight, was retracted as it was found no longer health protective (WHO 2011). What is more, due to available scientific data, it was not possible to establish a new PTWI for Pb (EFSA 2010; WHO 2010).

The average Pb level in a 200-mL beverage amounted to 0.002 mg/200 mL, which means that one cup of green tea provided 0.11 % of PTWI for Pb, suggesting that consumption of green tea does not exceed the former PTWI recommendation for this metal. In case of Cd, the mean content of this element in 200-mL beverage was under the detection limits of the method applied (Cd < 0.003 mg/100 g); therefore, it was concluded that there is no health risk associated with green tea infusion consumption.

The RDAs for the different elements were calculated for green tea infusions according to the latest available Polish (Jarosz 2012) and American recommendations (American nutritional standards 2011). One cup of tea per day provide approximately 1 % of the RDA for Ca, K, Mg, Na, and P, suggesting that green tea is not a rich source of macroelements in the daily diet (Table 5). Iron, Zn, Cu, and Ni provided approximately 1 % of the RDA. However, green tea can be a good source of Mn. The percentage of the RDA provided by one daily cup of tea was approximately 28.3 % for Mn, which is significant; however, its bioavailability to the human body needs to be considered. According to Powell et al. (1998), almost 40 % of Mn is bioavailable under simulated intestinal conditions, indicating that the RDA for Mn is reached in approximately 11.3 % of cases through consumption of five cups of green tea. The highest RDA realization through consumption of one cup daily was recorded for Cr (2.86–4.00 %). Seenivasan et al. (2008a, b) suggested that tea contamination by Cr originates mainly from soil but is also a result of CTC (crush, tear, curl) roller usage during tea processing. Mossion et al. (2008) implied that the percentage of elements leaching to infusions as well as the final composition of tea infusions is strongly dependent on water composition. Additionally, tea contains many anti-nutritive substances such as oxalate, which can absorb metals in solution. Taken together, our results suggest that drinking an average of five cups of green tea per day will not exceed the RDAs for the analyzed elements.

**Table 5:** Realization of recommended dietary intake through consumption of 1 cup of 200 mL green tea beverage

Element	Recommended daily allowance (RDA) [mg/day/person]		Average content (mg/200 mL)	Realization of RDA through consumption of 200 mL of infusion [%]	
	Males	Females		Males	Females
	(31–50 years)	(31–50 years)		(31–50 years)	(31–50 years)
Ca	1000	1000	0.35 ± 0.34	0.03	0.03
			<LOD–1.54		
K	4700	4700	23.4 ± 9.08	0.50	0.50
			8.57–60.6		
Mg	420	320	1.65 ± 0.48	0.39	0.51
			0.85–2.72		
Na	1500	1500	0.03 ± 001	0.002	0.002
			<LOD–0.05		
P	700	700	8.5 ± 3.7	1.21	1.21
			1.3–16		
Mn^a^	2.3	1.8	0.51 ± 0.15	22.2	28.3
			0.18–0.86		
Fe^b^	10	18	0.04 ± 0.006	0.40	0.22
			0.04–0.07		
Zn	11	8	0.03 ± 0.01	0.27	0.37
			<LOD–0.07		
Cu	0.9	0.9	0.01 ± 0.002	1.11	1.11
			<LOD–0.01		
Cr	0.035	0.025	0.001 ± 0.0001	2.86	4.00
			<LOD–0.001		
Ni	1	1	0.005 ± 0.002	0.5	0.5
			<LOD–0.01		

LOD for Ca = 0.02 mg/100 g; LOD for Na = 0.02 mg/100 g; LOD for Zn = 0.02 mg/100 g; LOD for Cu = 0.009 mg/100 g; LOD for Cr = 0.001 mg/100 g; LOD for Ni = 0.002 mg/100 g

^a^ American recommendations (American nutritional standards 2011)

^b^ Polish recommendations (Jarosz 2012)

## Conclusions

Assessment of the mineral composition of green tea is helpful to estimate the intake of toxic metals and bioelements associated with the consumption of infusions. Based on the FAO/WHO recommendations, we showed that consumption of green tea from China, Japan, India, and marketed tea is not associated with health hazards related to exposure to heavy metals such as Cd. Consumption of five cups of green tea per week does not pose a risk to human health. Comparison with the recent standards showed that green tea is characterized by high Mn levels.

The results of the present study suggest that chemometric techniques are very helpful tools for tea analysis and authenticity evaluation. These techniques enabled the differentiation of products according to geographical origin. Potassium, P, Mn, Fe, Cu, Co, and Cd were found to be appropriate descriptors of tea samples’ origin identification.
